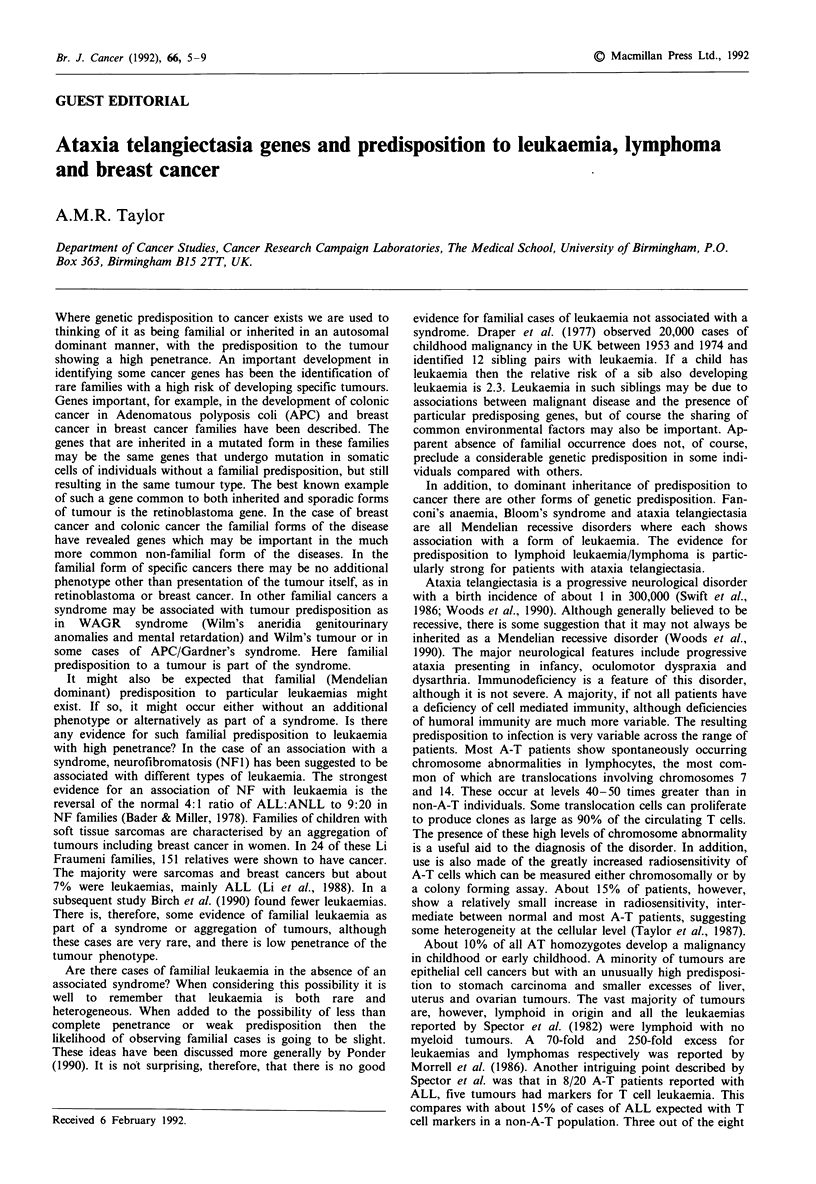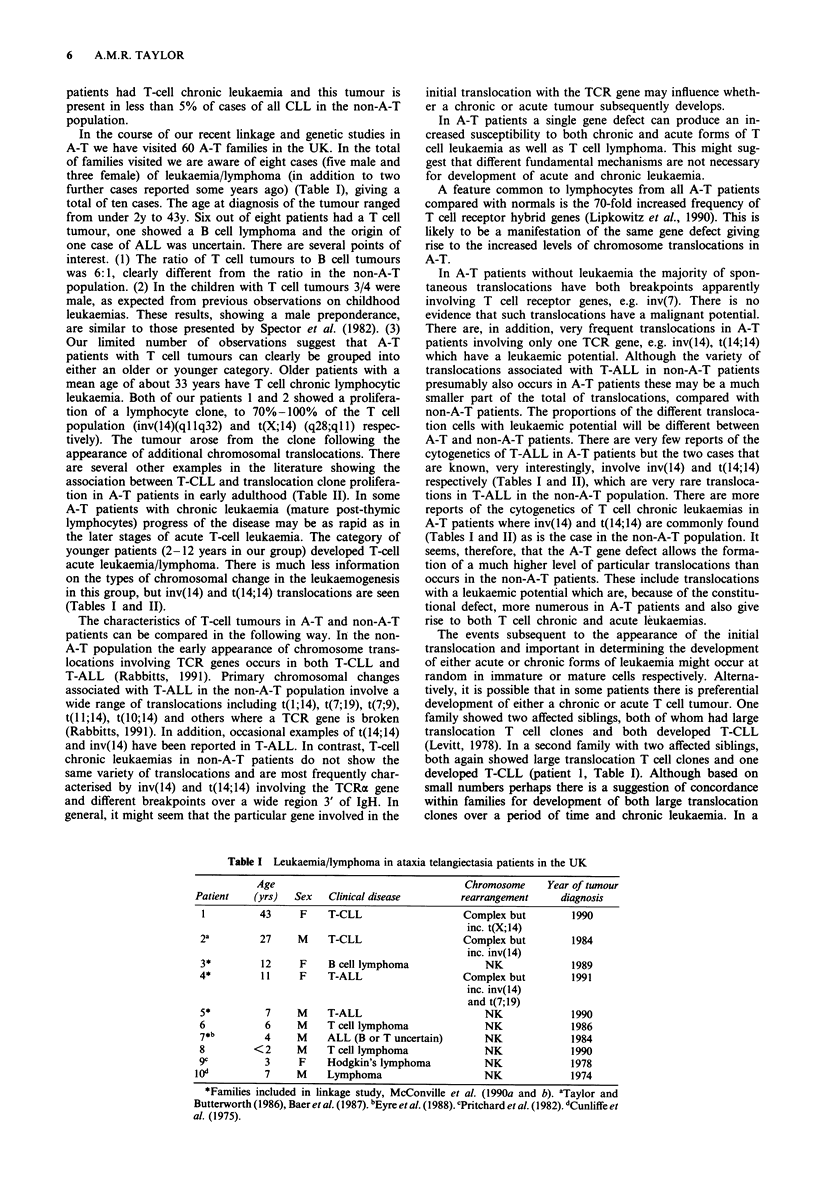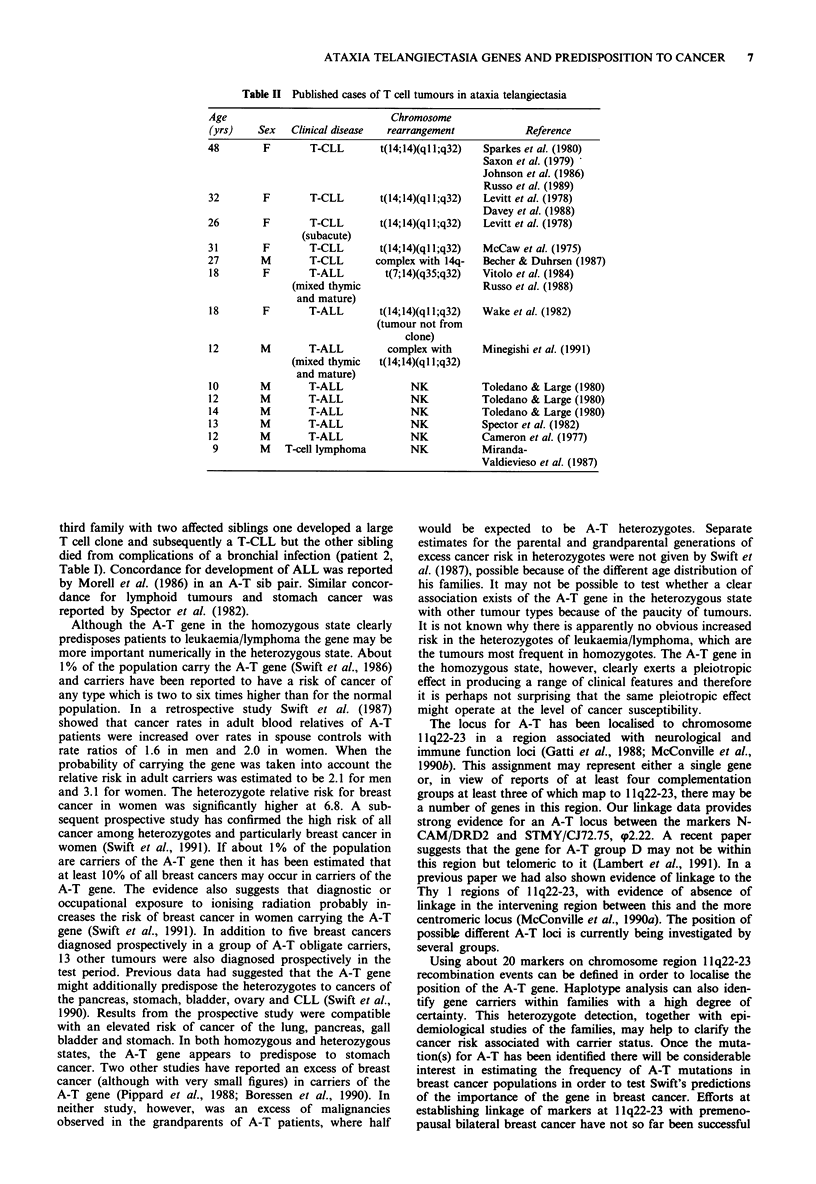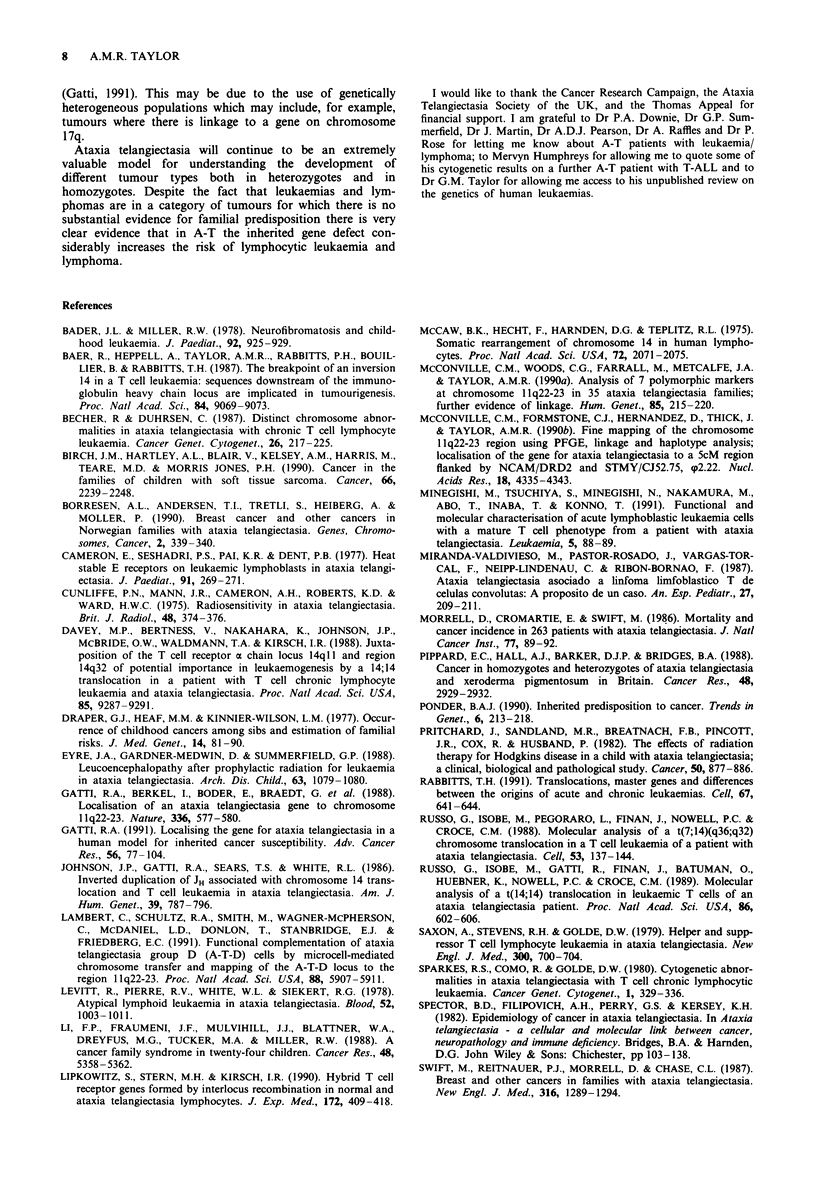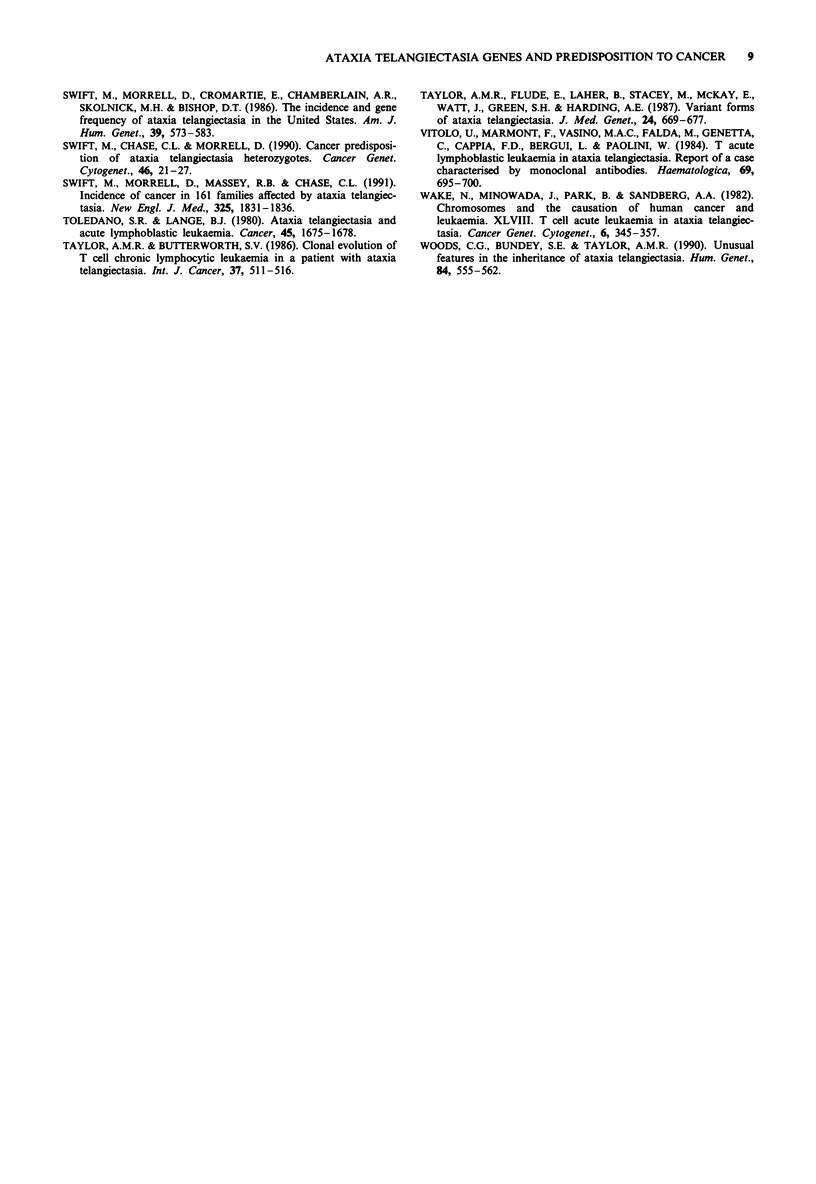# Ataxia telangiectasia genes and predisposition to leukaemia, lymphoma and breast cancer.

**DOI:** 10.1038/bjc.1992.208

**Published:** 1992-07

**Authors:** A. M. Taylor


Br. J. Cancer (1992), 66, 5–9 © Macmillan Press Ltd., 1992

GUEST EDITORIAL

### Ataxia telangiectasia genes and predisposition to leukaemia, lymphoma and breast cancer

A.M.R. Taylor

*Department of Cancer Studies, Cancer Research Campaign Laboratories, The Medical School, University of Birmingham, P.O. Box 363, Birmingham B15 2TT, UK.*

Where genetic predisposition to cancer exists we are used to thinking of it as being familial or inherited in an autosomal dominant manner, with the predisposition to the tumour showing a high penetrance. An important development in identifying some cancer genes has been the identification of rare families with a high risk of developing specific tumours. Genes important, for example, in the development of colonic cancer in Adenomatous polyposis coli (APC) and breast cancer in breast cancer families have been described. The genes that are inherited in a mutated form in these families may be the same genes that undergo mutation in somatic cells of individuals without a familial predisposition, but still resulting in the same tumour type. The best known example of such a gene common to both inherited and sporadic forms of tumour is the retinoblastoma gene. In the case of breast cancer and colonic cancer the familial forms of the disease have revealed genes which may be important in the much more common non-familial form of the diseases. In the familial form of specific cancers there may be no additional phenotype other than presentation of the tumour itself, as in retinoblastoma or breast cancer. In other familial cancers a syndrome may be associated with tumour predisposition as in WAGR syndrome (Wilm's aneridia genitourinary anomalies and mental retardation) and Wilm's tumour or in some cases of APC/Gardner's syndrome. Here familial predisposition to a tumour is part of the syndrome.

It might also be expected that familial (Mendelian dominant) predisposition to particular leukaemias might exist. If so, it might occur either without an additional phenotype or alternatively as part of a syndrome. Is there any evidence for such familial predisposition to leukaemia with high penetrance? In the case of an association with a syndrome, neurofibromatosis (NF1) has been suggested to be associated with different types of leukaemia. The strongest evidence for an association of NF with leukaemia is the reversal of the normal 4:1 ratio of ALL:ANLL to 9:20 in NF families (Bader & Miller, 1978). Families of children with soft tissue sarcomas are characterised by an aggregation of tumours including breast cancer in women. In 24 of these Li Fraumeni families, 151 relatives were shown to have cancer. The majority were sarcomas and breast cancers but about 7% were leukaemias, mainly ALL (Li *et al.*, 1988). In a subsequent study Birch *et al.* (1990) found fewer leukaemias. There is, therefore, some evidence of familial leukaemia as part of a syndrome or aggregation of tumours, although these cases are very rare, and there is low penetrance of the tumour phenotype.

Are there cases of familial leukaemia in the absence of an associated syndrome? When considering this possibility it is well to remember that leukaemia is both rare and heterogeneous. When added to the possibility of less than complete penetrance or weak predisposition then the likelihood of observing familial cases is going to be slight. These ideas have been discussed more generally by Ponder (1990). It is not surprising, therefore, that there is no good evidence for familial cases of leukaemia not associated with a syndrome. Draper *et al.* (1977) observed 20,000 cases of childhood malignancy in the UK between 1953 and 1974 and identified 12 sibling pairs with leukaemia. If a child has leukaemia then the relative risk of a sib also developing leukaemia is 2.3. Leukaemia in such siblings may be due to associations between malignant disease and the presence of particular predisposing genes, but of course the sharing of common environmental factors may also be important. Apparent absence of familial occurrence does not, of course, preclude a considerable genetic predisposition in some individuals compared with others.

In addition, to dominant inheritance of predisposition to cancer there are other forms of genetic predisposition. Fanconi's anaemia, Bloom's syndrome and ataxia telangiectasia are all Mendelian recessive disorders where each shows association with a form of leukaemia. The evidence for predisposition to lymphoid leukaemia/lymphoma is particularly strong for patients with ataxia telangiectasia.

Ataxia telangiectasia is a progressive neurological disorder with a birth incidence of about 1 in 300,000 (Swift *et al.*, 1986; Woods *et al.*, 1990). Although generally believed to be recessive, there is some suggestion that it may not always be inherited as a Mendelian recessive disorder (Woods *et al.*, 1990). The major neurological features include progressive ataxia presenting in infancy, oculomotor dyspraxia and dysarthria. Immunodeficiency is a feature of this disorder, although it is not severe. A majority, if not all patients have a deficiency of cell mediated immunity, although deficiencies of humoral immunity are much more variable. The resulting predisposition to infection is very variable across the range of patients. Most A-T patients show spontaneously occurring chromosome abnormalities in lymphocytes, the most common of which are translocations involving chromosomes 7 and 14. These occur at levels 40–50 times greater than in non-A-T individuals. Some translocation cells can proliferate to produce clones as large as 90% of the circulating T cells. The presence of these high levels of chromosome abnormality is a useful aid to the diagnosis of the disorder. In addition, use is also made of the greatly increased radiosensitivity of A-T cells which can be measured either chromosomally or by a colony forming assay. About 15% of patients, however, show a relatively small increase in radiosensitivity, intermediate between normal and most A-T patients, suggesting some heterogeneity at the cellular level (Taylor *et al.*, 1987).

About 10% of all AT homozygotes develop a malignancy in childhood or early childhood. A minority of tumours are epithelial cell cancers but with an unusually high predisposition to stomach carcinoma and smaller excesses of liver, uterus and ovarian tumours. The vast majority of tumours are, however, lymphoid in origin and all the leukaemias reported by Spector *et al.* (1982) were lymphoid with no myeloid tumours. A 70-fold and 250-fold excess for leukaemias and lymphomas respectively was reported by Morrell *et al.* (1986). Another intriguing point described by Spector *et al.* was that in 8/20 A-T patients reported with ALL, five tumours had markers for T cell leukaemia. This compares with about 15% of cases of ALL expected with T cell markers in a non-A-T population. Three out of the eight

Received 6 February 1992.

patients had T-cell chronic leukaemia and this tumour is present in less than 5% of cases of all CLL in the non-A-T population.

In the course of our recent linkage and genetic studies in A-T we have visited 60 A-T families in the UK. In the total of families visited we are aware of eight cases (five male and three female) of leukaemia/lymphoma (in addition to two further cases reported some years ago) (Table I), giving a total of ten cases. The age at diagnosis of the tumour ranged from under 2y to 43y. Six out of eight patients had a T cell tumour, one showed a B cell lymphoma and the origin of one case of ALL was uncertain. There are several points of interest. (1) The ratio of T cell tumours to B cell tumours was 6:1, clearly different from the ratio in the non-A-T population. (2) In the children with T cell tumours 3/4 were male, as expected from previous observations on childhood leukaemias. These results, showing a male preponderance, are similar to those presented by Spector *et al.* (1982). (3) Our limited number of observations suggest that A-T patients with T cell tumours can clearly be grouped into either an older or younger category. Older patients with a mean age of about 33 years have T cell chronic lymphocytic leukaemia. Both of our patients 1 and 2 showed a proliferation of a lymphocyte clone, to 70%–100% of the T cell population (inv(14)(q11q32) and t(X;14) (q28;q11) respectively). The tumour arose from the clone following the appearance of additional chromosomal translocations. There are several other examples in the literature showing the association between T-CLL and translocation clone proliferation in A-T patients in early adulthood (Table II). In some A-T patients with chronic leukaemia (mature post-thymic lymphocytes) progress of the disease may be as rapid as in the later stages of acute T-cell leukaemia. The category of younger patients (2–12 years in our group) developed T-cell acute leukaemia/lymphoma. There is much less information on the types of chromosomal change in the leukaemogenesis in this group, but inv(14) and t(14;14) translocations are seen (Tables I and II).

The characteristics of T-cell tumours in A-T and non-A-T patients can be compared in the following way. In the non-A-T population the early appearance of chromosome translocations involving TCR genes occurs in both T-CLL and T-ALL (Rabbitts, 1991). Primary chromosomal changes associated with T-ALL in the non-A-T population involve a wide range of translocations including t(1;14), t(7;19), t(7;9), t(11;14), t(10;14) and others where a TCR gene is broken (Rabbitts, 1991). In addition, occasional examples of t(14;14) and inv(14) have been reported in T-ALL. In contrast, T-cell chronic leukaemias in non-A-T patients do not show the same variety of translocations and are most frequently characterised by inv(14) and t(14;14) involving the TCRα gene and different breakpoints over a wide region 3′ of IgH. In general, it might seem that the particular gene involved in the initial translocation with the TCR gene may influence whether a chronic or acute tumour subsequently develops.

In A-T patients a single gene defect can produce an increased susceptibility to both chronic and acute forms of T cell leukaemia as well as T cell lymphoma. This might suggest that different fundamental mechanisms are not necessary for development of acute and chronic leukaemia.

A feature common to lymphocytes from all A-T patients compared with normals is the 70-fold increased frequency of T cell receptor hybrid genes (Lipkowitz *et al.*, 1990). This is likely to be a manifestation of the same gene defect giving rise to the increased levels of chromosome translocations in A-T.

In A-T patients without leukaemia the majority of spontaneous translocations have both breakpoints apparently involving T cell receptor genes, e.g. inv(7). There is no evidence that such translocations have a malignant potential. There are, in addition, very frequent translocations in A-T patients involving only one TCR gene, e.g. inv(14), t(14;14) which have a leukaemic potential. Although the variety of translocations associated with T-ALL in non-A-T patients presumably also occurs in A-T patients these may be a much smaller part of the total of translocations, compared with non-A-T patients. The proportions of the different translocation cells with leukaemic potential will be different between A-T and non-A-T patients. There are very few reports of the cytogenetics of T-ALL in A-T patients but the two cases that are known, very interestingly, involve inv(14) and t(14;14) respectively (Tables I and II), which are very rare translocations in T-ALL in the non-A-T population. There are more reports of the cytogenetics of T cell chronic leukaemias in A-T patients where inv(14) and t(14;14) are commonly found (Tables I and II) as is the case in the non-A-T population. It seems, therefore, that the A-T gene defect allows the formation of a much higher level of particular translocations than occurs in the non-A-T patients. These include translocations with a leukaemic potential which are, because of the constitutional defect, more numerous in A-T patients and also give rise to both T cell chronic and acute leukaemias.

The events subsequent to the appearance of the initial translocation and important in determining the development of either acute or chronic forms of leukaemia might occur at random in immature or mature cells respectively. Alternatively, it is possible that in some patients there is preferential development of either a chronic or acute T cell tumour. One family showed two affected siblings, both of whom had large translocation T cell clones and both developed T-CLL (Levitt, 1978). In a second family with two affected siblings, both again showed large translocation T cell clones and one developed T-CLL (patient 1, Table I). Although based on small numbers perhaps there is a suggestion of concordance within families for development of both large translocation clones over a period of time and chronic leukaemia. In a

**Table I** Leukaemia/lymphoma in ataxia telangiectasia patients in the UK

| *Patient* | *Age (yrs)* | *Sex* | *Clinical disease* | *Chromosome rearrangement* | *Year of tumour diagnosis* |
|---|---|---|---|---|---|
| 1 | 43 | F | T-CLL | Complex but inc. t(X;14) | 1990 |
| 2[a] | 27 | M | T-CLL | Complex but inc. inv(14) | 1984 |
| 3* | 12 | F | B cell lymphoma | NK | 1989 |
| 4* | 11 | F | T-ALL | Complex but inc. inv(14) and t(7;19) | 1991 |
| 5* | 7 | M | T-ALL | NK | 1990 |
| 6 | 6 | M | T cell lymphoma | NK | 1986 |
| 7*[b] | 4 | M | ALL (B or T uncertain) | NK | 1984 |
| 8 | <2 | M | T cell lymphoma | NK | 1990 |
| 9[c] | 3 | F | Hodgkin's lymphoma | NK | 1978 |
| 10[d] | 7 | M | Lymphoma | NK | 1974 |

*Families included in linkage study, McConville *et al.* (1990*a* and *b*). [a]Taylor and Butterworth (1986), Baer *et al.* (1987). [b]Eyre *et al.* (1988). [c]Pritchard *et al.* (1982). [d]Cunliffe *et al.* (1975).

**Table II** Published cases of T cell tumours in ataxia telangiectasia

| *Age (yrs)* | *Sex* | *Clinical disease* | *Chromosome rearrangement* | *Reference* |
|---|---|---|---|---|
| 48 | F | T-CLL | t(14;14)(q11;q32) | Sparkes *et al.* (1980)<br>Saxon *et al.* (1979)<br>Johnson *et al.* (1986)<br>Russo *et al.* (1989) |
| 32 | F | T-CLL | t(14;14)(q11;q32) | Levitt *et al.* (1978)<br>Davey *et al.* (1988) |
| 26 | F | T-CLL (subacute) | t(14;14)(q11;q32) | Levitt *et al.* (1978) |
| 31 | F | T-CLL | t(14;14)(q11;q32) | McCaw *et al.* (1975) |
| 27 | M | T-CLL | complex with 14q- | Becher & Duhrsen (1987) |
| 18 | F | T-ALL (mixed thymic and mature) | t(7;14)(q35;q32) | Vitolo *et al.* (1984)<br>Russo *et al.* (1988) |
| 18 | F | T-ALL | t(14;14)(q11;q32) (tumour not from clone) | Wake *et al.* (1982) |
| 12 | M | T-ALL (mixed thymic and mature) | complex with t(14;14)(q11;q32) | Minegishi *et al.* (1991) |
| 10 | M | T-ALL | NK | Toledano & Large (1980) |
| 12 | M | T-ALL | NK | Toledano & Large (1980) |
| 14 | M | T-ALL | NK | Toledano & Large (1980) |
| 13 | M | T-ALL | NK | Spector *et al.* (1982) |
| 12 | M | T-ALL | NK | Cameron *et al.* (1977) |
| 9 | M | T-cell lymphoma | NK | Miranda-Valdievieso *et al.* (1987) |

third family with two affected siblings one developed a large T cell clone and subsequently a T-CLL but the other sibling died from complications of a bronchial infection (patient 2, Table I). Concordance for development of ALL was reported by Morell *et al.* (1986) in an A-T sib pair. Similar concordance for lymphoid tumours and stomach cancer was reported by Spector *et al.* (1982).

Although the A-T gene in the homozygous state clearly predisposes patients to leukaemia/lymphoma the gene may be more important numerically in the heterozygous state. About 1% of the population carry the A-T gene (Swift *et al.*, 1986) and carriers have been reported to have a risk of cancer of any type which is two to six times higher than for the normal population. In a retrospective study Swift *et al.* (1987) showed that cancer rates in adult blood relatives of A-T patients were increased over rates in spouse controls with rate ratios of 1.6 in men and 2.0 in women. When the probability of carrying the gene was taken into account the relative risk in adult carriers was estimated to be 2.1 for men and 3.1 for women. The heterozygote relative risk for breast cancer in women was significantly higher at 6.8. A subsequent prospective study has confirmed the high risk of all cancer among heterozygotes and particularly breast cancer in women (Swift *et al.*, 1991). If about 1% of the population are carriers of the A-T gene then it has been estimated that at least 10% of all breast cancers may occur in carriers of the A-T gene. The evidence also suggests that diagnostic or occupational exposure to ionising radiation probably increases the risk of breast cancer in women carrying the A-T gene (Swift *et al.*, 1991). In addition to five breast cancers diagnosed prospectively in a group of A-T obligate carriers, 13 other tumours were also diagnosed prospectively in the test period. Previous data had suggested that the A-T gene might additionally predispose the heterozygotes to cancers of the pancreas, stomach, bladder, ovary and CLL (Swift *et al.*, 1990). Results from the prospective study were compatible with an elevated risk of cancer of the lung, pancreas, gall bladder and stomach. In both homozygous and heterozygous states, the A-T gene appears to predispose to stomach cancer. Two other studies have reported an excess of breast cancer (although with very small figures) in carriers of the A-T gene (Pippard *et al.*, 1988; Boressen *et al.*, 1990). In neither study, however, was an excess of malignancies observed in the grandparents of A-T patients, where half would be expected to be A-T heterozygotes. Separate estimates for the parental and grandparental generations of excess cancer risk in heterozygotes were not given by Swift *et al.* (1987), possible because of the different age distribution of his families. It may not be possible to test whether a clear association exists of the A-T gene in the heterozygous state with other tumour types because of the paucity of tumours. It is not known why there is apparently no obvious increased risk in the heterozygotes of leukaemia/lymphoma, which are the tumours most frequent in homozygotes. The A-T gene in the homozygous state, however, clearly exerts a pleiotropic effect in producing a range of clinical features and therefore it is perhaps not surprising that the same pleiotropic effect might operate at the level of cancer susceptibility.

The locus for A-T has been localised to chromosome 11q22-23 in a region associated with neurological and immune function loci (Gatti *et al.*, 1988; McConville *et al.*, 1990*b*). This assignment may represent either a single gene or, in view of reports of at least four complementation groups at least three of which map to 11q22-23, there may be a number of genes in this region. Our linkage data provides strong evidence for an A-T locus between the markers N-CAM/DRD2 and STMY/CJ72.75, φ2.22. A recent paper suggests that the gene for A-T group D may not be within this region but telomeric to it (Lambert *et al.*, 1991). In a previous paper we had also shown evidence of linkage to the Thy 1 regions of 11q22-23, with evidence of absence of linkage in the intervening region between this and the more centromeric locus (McConville *et al.*, 1990*a*). The position of possible different A-T loci is currently being investigated by several groups.

Using about 20 markers on chromosome region 11q22-23 recombination events can be defined in order to localise the position of the A-T gene. Haplotype analysis can also identify gene carriers within families with a high degree of certainty. This heterozygote detection, together with epidemiological studies of the families, may help to clarify the cancer risk associated with carrier status. Once the mutation(s) for A-T has been identified there will be considerable interest in estimating the frequency of A-T mutations in breast cancer populations in order to test Swift's predictions of the importance of the gene in breast cancer. Efforts at establishing linkage of markers at 11q22-23 with premenopausal bilateral breast cancer have not so far been successful

(Gatti, 1991). This may be due to the use of genetically heterogeneous populations which may include, for example, tumours where there is linkage to a gene on chromosome 17q.

Ataxia telangiectasia will continue to be an extremely valuable model for understanding the development of different tumour types both in heterozygotes and in homozygotes. Despite the fact that leukaemias and lymphomas are in a category of tumours for which there is no substantial evidence for familial predisposition there is very clear evidence that in A-T the inherited gene defect considerably increases the risk of lymphocytic leukaemia and lymphoma.

I would like to thank the Cancer Research Campaign, the Ataxia Telangiectasia Society of the UK, and the Thomas Appeal for financial support. I am grateful to Dr P.A. Downie, Dr G.P. Summerfield, Dr J. Martin, Dr A.D.J. Pearson, Dr A. Raffles and Dr P. Rose for letting me know about A-T patients with leukaemia/lymphoma; to Mervyn Humphreys for allowing me to quote some of his cytogenetic results on a further A-T patient with T-ALL and to Dr G.M. Taylor for allowing me access to his unpublished review on the genetics of human leukaemias.

### References

BADER, J.L. & MILLER, R.W. (1978). Neurofibromatosis and childhood leukaemia. *J. Paediat.*, **92**, 925–929.

BAER, R., HEPPELL, A., TAYLOR, A.M.R.., RABBITTS, P.H., BOUILLIER, B. & RABBITTS, T.H. (1987). The breakpoint of an inversion 14 in a T cell leukaemia: sequences downstream of the immunoglobulin heavy chain locus are implicated in tumourigenesis. *Proc. Natl Acad. Sci.*, **84**, 9069–9073.

BECHER, R & DUHRSEN, C. (1987). Distinct chromosome abnormalities in ataxia telangiectasia with chronic T cell lymphocyte leukaemia. *Cancer Genet. Cytogenet.*, **26**, 217–225.

BIRCH, J.M., HARTLEY, A.L., BLAIR, V., KELSEY, A.M., HARRIS, M., TEARE, M.D. & MORRIS JONES, P.H. (1990). Cancer in the families of children with soft tissue sarcoma. *Cancer*, **66**, 2239–2248.

BORRESEN, A.L., ANDERSEN, T.I., TRETLI, S., HEIBERG, A. & MOLLER, P. (1990). Breast cancer and other cancers in Norwegian families with ataxia telangiectasia. *Genes, Chromosomes, Cancer*, **2**, 339–340.

CAMERON, E., SESHADRI, P.S., PAI, K.R. & DENT, P.B. (1977). Heat stable E receptors on leukaemic lymphoblasts in ataxia telangiectasia. *J. Paediat.*, **91**, 269–271.

CUNLIFFE, P.N., MANN, J.R., CAMERON, A.H., ROBERTS, K.D. & WARD, H.W.C. (1975). Radiosensitivity in ataxia telangiectasia. *Brit. J. Radiol.*, **48**, 374–376.

DAVEY, M.P., BERTNESS, V., NAKAHARA, K., JOHNSON, J.P., MCBRIDE, O.W., WALDMANN, T.A. & KIRSCH, I.R. (1988). Juxtaposition of the T cell receptor α chain locus 14q11 and region 14q32 of potential importance in leukaemogenesis by a 14;14 translocation in a patient with T cell chronic lymphocyte leukaemia and ataxia telangiectasia. *Proc. Natl Acad. Sci. USA*, **85**, 9287–9291.

DRAPER, G.J., HEAF, M.M. & KINNIER-WILSON, L.M. (1977). Occurrence of childhood cancers among sibs and estimation of familial risks. *J. Med. Genet.*, **14**, 81–90.

EYRE, J.A., GARDNER-MEDWIN, D. & SUMMERFIELD, G.P. (1988). Leucoencephalopathy after prophylactic radiation for leukaemia in ataxia telangiectasia. *Arch. Dis. Child.*, **63**, 1079–1080.

GATTI, R.A., BERKEL, I., BODER, E., BRAEDT, G. *et al.* (1988). Localisation of an ataxia telangiectasia gene to chromosome 11q22-23. *Nature*, **336**, 577–580.

GATTI, R.A. (1991). Localising the gene for ataxia telangiectasia in a human model for inherited cancer susceptibility. *Adv. Cancer Res.*, **56**, 77–104.

JOHNSON, J.P., GATTI, R.A., SEARS, T.S. & WHITE, R.L. (1986). Inverted duplication of $J_H$ associated with chromosome 14 translocation and T cell leukaemia in ataxia telangiectasia. *Am. J. Hum. Genet.*, **39**, 787–796.

LAMBERT, C., SCHULTZ, R.A., SMITH, M., WAGNER-MCPHERSON, C., MCDANIEL, L.D., DONLON, T., STANBRIDGE, E.J. & FRIEDBERG, E.C. (1991). Functional complementation of ataxia telangiectasia group D (A-T-D) cells by microcell-mediated chromosome transfer and mapping of the A-T-D locus to the region 11q22-23. *Proc. Natl Acad. Sci. USA*, **88**, 5907–5911.

LEVITT, R., PIERRE, R.V., WHITE, W.L. & SIEKERT, R.G. (1978). Atypical lymphoid leukaemia in ataxia telangiectasia. *Blood*, **52**, 1003–1011.

LI, F.P., FRAUMENI, J.F., MULVIHILL, J.J., BLATTNER, W.A., DREYFUS, M.G., TUCKER, M.A. & MILLER, R.W. (1988). A cancer family syndrome in twenty-four children. *Cancer Res.*, **48**, 5358–5362.

LIPKOWITZ, S., STERN, M.H. & KIRSCH, I.R. (1990). Hybrid T cell receptor genes formed by interlocus recombination in normal and ataxia telangiectasia lymphocytes. *J. Exp. Med.*, **172**, 409–418.

MCCAW, B.K., HECHT, F., HARNDEN, D.G. & TEPLITZ, R.L. (1975). Somatic rearrangement of chromosome 14 in human lymphocytes. *Proc. Natl Acad. Sci. USA*, **72**, 2071–2075.

MCCONVILLE, C.M., WOODS, C.G., FARRALL, M., METCALFE, J.A. & TAYLOR, A.M.R. (1990*a*). Analysis of 7 polymorphic markers at chromosome 11q22-23 in 35 ataxia telangiectasia families; further evidence of linkage. *Hum. Genet.*, **85**, 215–220.

MCCONVILLE, C.M., FORMSTONE, C.J., HERNANDEZ, D., THICK, J. & TAYLOR, A.M.R. (1990*b*). Fine mapping of the chromosome 11q22-23 region using PFGE, linkage and haplotype analysis; localisation of the gene for ataxia telangiectasia to a 5cM region flanked by NCAM/DRD2 and STMY/CJ52.75, φ2.22. *Nucl. Acids Res.*, **18**, 4335–4343.

MINEGISHI, M., TSUCHIYA, S., MINEGISHI, N., NAKAMURA, M., ABO, T., INABA, T. & KONNO, T. (1991). Functional and molecular characterisation of acute lymphoblastic leukaemia cells with a mature T cell phenotype from a patient with ataxia telangiectasia. *Leukaemia*, **5**, 88–89.

MIRANDA-VALDIVIESO, M., PASTOR-ROSADO, J., VARGAS-TORCAL, F., NEIPP-LINDENAU, C. & RIBON-BORNAO, F. (1987). Ataxia telangiectasia asociado a linfoma limfoblastico T de celulas convolutas: A proposito de un caso. *An. Esp. Pediatr.*, **27**, 209–211.

MORRELL, D., CROMARTIE, E. & SWIFT, M. (1986). Mortality and cancer incidence in 263 patients with ataxia telangiectasia. *J. Natl Cancer Inst.*, **77**, 89–92.

PIPPARD, E.C., HALL, A.J., BARKER, D.J.P. & BRIDGES, B.A. (1988). Cancer in homozygotes and heterozygotes of ataxia telangiectasia and xeroderma pigmentosum in Britain. *Cancer Res.*, **48**, 2929–2932.

PONDER, B.A.J. (1990). Inherited predisposition to cancer. *Trends in Genet.*, **6**, 213–218.

PRITCHARD, J., SANDLAND, M.R., BREATNACH, F.B., PINCOTT, J.R., COX, R. & HUSBAND, P. (1982). The effects of radiation therapy for Hodgkins disease in a child with ataxia telangiectasia; a clinical, biological and pathological study. *Cancer*, **50**, 877–886.

RABBITTS, T.H. (1991). Translocations, master genes and differences between the origins of acute and chronic leukaemias. *Cell*, **67**, 641–644.

RUSSO, G., ISOBE, M., PEGORARO, L., FINAN, J., NOWELL, P.C. & CROCE, C.M. (1988). Molecular analysis of a t(7;14)(q36;q32) chromosome translocation in a T cell leukaemia of a patient with ataxia telangiectasia. *Cell*, **53**, 137–144.

RUSSO, G., ISOBE, M., GATTI, R., FINAN, J., BATUMAN, O., HUEBNER, K., NOWELL, P.C. & CROCE, C.M. (1989). Molecular analysis of a t(14;14) translocation in leukaemic T cells of an ataxia telangiectasia patient. *Proc. Natl Acad. Sci. USA*, **86**, 602–606.

SAXON, A., STEVENS, R.H. & GOLDE, D.W. (1979). Helper and suppressor T cell lymphocyte leukaemia in ataxia telangiectasia. *New Engl. J. Med.*, **300**, 700–704.

SPARKES, R.S., COMO, R. & GOLDE, D.W. (1980). Cytogenetic abnormalities in ataxia telangiectasia with T cell chronic lymphocytic leukaemia. *Cancer Genet. Cytogenet.*, **1**, 329–336.

SPECTOR, B.D., FILIPOVICH, A.H., PERRY, G.S. & KERSEY, K.H. (1982). Epidemiology of cancer in ataxia telangiectasia. In *Ataxia telangiectasia - a cellular and molecular link between cancer, neuropathology and immune deficiency*. Bridges, B.A. & Harnden, D.G. John Wiley & Sons: Chichester, pp 103–138.

SWIFT, M., REITNAUER, P.J., MORRELL, D. & CHASE, C.L. (1987). Breast and other cancers in families with ataxia telangiectasia. *New Engl. J. Med.*, **316**, 1289–1294.

SWIFT, M., MORRELL, D., CROMARTIE, E., CHAMBERLAIN, A.R., SKOLNICK, M.H. & BISHOP, D.T. (1986). The incidence and gene frequency of ataxia telangiectasia in the United States. *Am. J. Hum. Genet.*, **39**, 573-583.

SWIFT, M., CHASE, C.L. & MORRELL, D. (1990). Cancer predisposition of ataxia telangiectasia heterozygotes. *Cancer Genet. Cytogenet.*, **46**, 21-27.

SWIFT, M., MORRELL, D., MASSEY, R.B. & CHASE, C.L. (1991). Incidence of cancer in 161 families affected by ataxia telangiectasia. *New Engl. J. Med.*, **325**, 1831-1836.

TOLEDANO, S.R. & LANGE, B.J. (1980). Ataxia telangiectasia and acute lymphoblastic leukaemia. *Cancer*, **45**, 1675-1678.

TAYLOR, A.M.R. & BUTTERWORTH, S.V. (1986). Clonal evolution of T cell chronic lymphocytic leukaemia in a patient with ataxia telangiectasia. *Int. J. Cancer*, **37**, 511-516.

TAYLOR, A.M.R., FLUDE, E., LAHER, B., STACEY, M., MCKAY, E., WATT, J., GREEN, S.H. & HARDING, A.E. (1987). Variant forms of ataxia telangiectasia. *J. Med. Genet.*, **24**, 669-677.

VITOLO, U., MARMONT, F., VASINO, M.A.C., FALDA, M., GENETTA, C., CAPPIA, F.D., BERGUI, L. & PAOLINI, W. (1984). T acute lymphoblastic leukaemia in ataxia telangiectasia. Report of a case characterised by monoclonal antibodies. *Haematologica*, **69**, 695-700.

WAKE, N., MINOWADA, J., PARK, B. & SANDBERG, A.A. (1982). Chromosomes and the causation of human cancer and leukaemia. XLVIII. T cell acute leukaemia in ataxia telangiectasia. *Cancer Genet. Cytogenet.*, **6**, 345-357.

WOODS, C.G., BUNDEY, S.E. & TAYLOR, A.M.R. (1990). Unusual features in the inheritance of ataxia telangiectasia. *Hum. Genet.*, **84**, 555-562.